# Promoting Growth Performances and Phytochemicals of Black Upland Rice Through the Co-Inoculation of Arbuscular Mycorrhizal Fungi and Endophytic Fungi Under Drought Conditions

**DOI:** 10.3390/jof12010002

**Published:** 2025-12-19

**Authors:** Saralee Suphaphan, Thanawan Gateta, Wasan Seemakram, Thanapat Suebrasri, Saranya Chantawong, Chaiya Klinsukon, Piyada Theerakulpisut, Sophon Boonlue

**Affiliations:** 1 Department of Microbiology, Faculty of Science, Khon Kaen University, Khon Kaen 40002, Thailand; saralee.su@kkumail.com (S.S.); thanawan.gateta@kkumail.com (T.G.); 2 Department of Microbiology and Parasitology, Faculty of Medical Science, Naresuan University, Phitsanulok 65000, Thailand; wasans@nu.ac.th; 3 Division of Basic and Preclinical Science, Institute of Science and General Education, Nakhon Ratchasima College, Nakhon Ratchasima 30000, Thailand; s.thanapat@nmc.ac.th; 4 School of Preclinical Sciences, Institute of Science, Suranaree University of Technology, Nakhon Ratchasima 30000, Thailand; saranya.ch@sut.ac.th; 5 Department of Innovative Agriculture, Khon Kaen University, Khon Kaen 40002, Thailand; chaikl@kku.ac.th; 6 Department of Biology, Faculty of Science, Khon Kaen University, Khon Kaen 40002, Thailand; piythe@kku.ac.th

**Keywords:** anthocyanin, antioxidant, black rice, drought stress, Maled Phai rice, organic agriculture

## Abstract

Drought is a major problem affecting upland rice growth worldwide, including in northeast Thailand, with insufficient irrigation, where drought stress leads to reduced yields and may affect the functional compound content of rice grains. This research aimed to study the efficacy of arbuscular mycorrhizal fungi (AMF) *Rhizophagus variabilis* KS-02 and endophytic fungi (EPF) *Trichoderma zelobreve* PBMP16 on promoting the growth and accumulation of functional substances in upland black rice under drought conditions. Factorial experiments in a randomized complete block design (RCBD) were conducted by cultivating rice inoculated with AMF and EPF as well as co-inoculated with AMF+EPF under three watering conditions: 100% field capacity (FC), 66% FC, and 33% FC. The results show that both AMF, EPF improved some plant growth parameters and physiological performance under both well-watered and water-limited conditions. Inoculating plants with fungi increased the production of enzymes APX, CAT, and GR, as well as proline, which helps plants tolerate water deficit stress. Functional grain quality, including phenolic compounds, anthocyanins, and antioxidant activity, was also increased by fungal inoculation. While co-inoculation provided advantages for certain parameters, particularly antioxidant activity and biomass, single inoculation with AMF or EPF was equally effective or superior for specific traits depending on the level of water stress. Overall, this report shows that both AMF and EPF contribute to improving the productivity and functional quality of upland black rice under drought conditions, with treatment effects varying according to fungal type and water availability.

## 1. Introduction

Rice (*Oryza sativa* L.) is a globally popular agricultural crop, primarily produced and consumed in Asia, especially Thailand. According to Fukasawa and Ziska [1], rice is highly nutritious, serving as a major source of carbohydrates and providing essential minerals and vitamins. Especially noteworthy are the unique varieties of rice with red, purple, or black grains; the variety commonly referred to as black rice contains a higher level of bioactive compounds than regular white rice. In particular, these bioactive compounds include phenolic compounds and anthocyanins, which have antioxidant, anti-inflammatory, anti-diabetic, and anti-obesity properties [2,3]. Given its nutritional value, black rice has become increasingly popular in the market and is being promoted for production in several regions in Thailand, including the northeastern part of the country. However, black rice production in this region often has low yields because many of its cultivation areas face drought problems every year. Agricultural areas that rely on rainwater often experience water shortages, leading to poor growth of rice plants. In addition, the areas consist of sandy and sandy loam soils, which have low levels of cations. This leads to weak soil organic carbon connectivity, resulting in low water and nutrient retention in the soil [4,5]. Farmers widely use chemical fertilizers to increase crop yield and enrich soil nutrients [6]. However, chemical fertilizers affect soil quality, notably reducing beneficial soil microbes [7,8]. It is of interest to reduce synthetic fertilizer use and promote growth and seed yield through the use of plant growth-promoting microorganisms (PGPMs); PGPMs have been extensively studied due to their environmentally friendly nature [9].

This environmentally friendly approach enhances plant growth and yield even under drought stress, and examples of plant growth-promoting microorganisms (PGPMs) include arbuscular mycorrhizal fungi (AMF) and endophytic fungi (EPF) [10]. AMF develop extraradical mycelia that extend the depletion zone that develops around roots and facilitate the acquisition of low-mobility nutrients [11]. In addition, this fungus can produce certain glycoproteins that contribute to the aggregation of soil particles, enhancing the soil’s water retention capabilities. It has beneficial effects on the uptake of nutrients that help promote plant growth, such as phosphorus (P), nitrogen (N), potassium (K), and micronutrients [12]. According to the research by Tisarum et al. [13], under water-limited conditions, AMF enhance nutrient absorption for rice, promoting the growth of rice plants and reducing yield losses caused by drought stress. EPF can reside in plant cells, existing throughout the plant’s structure in leaves, stems, and roots. This is interesting because EPF play an essential role in plant growth promotion, leading to a higher yield through an increase in nutrient absorption, a reduction in disease severity, the improvement of host resistance against environmental stresses, and the production of secondary metabolites (phenols and flavonoids) [14,15]. There is also a tendency to increase photosynthetic activity and boost the accumulation of antioxidant compounds, including anthocyanins and phenolic compounds, in black rice [16]. Furthermore, rice seedlings that have been inoculated with EPF and grown under drought conditions tend to wilt more slowly and have higher chlorophyll content in their leaves compared to those not grown with the fungi. This suggests that EPF may positively promote the photosynthesis mechanism of rice under drought conditions while also maintaining the integrity of the rice grains [17,18].

In this study, species of AMF, *Rhizophagus variabilis* KS-02, and EPF, *Trichoderma zelobreve* PBMP16, were selected for use due to their ability to promote plant growth, yield, phenolic compounds, and anthocyanins in black upland rice [16,19]. Previous studies have primarily focused on the individual effects of AMF or EPF on rice performance. However, the synergistic effects between EPF and AMF on promoting plant growth, yield, phenolic compounds, and anthocyanins in upland black rice under drought stress have not been reported. Therefore, this work also aimed to investigate their promotion of the above properties individually and in co-inoculation in a pot trial. The results are expected to demonstrate that the application of AMF and EPF individually and in co-inoculation can promote rice plant growth and the phytochemicals of black rice under drought stress in Thailand.

## 2. Materials and Methods

### 2.1. Fungi

Species of AMF, *R. variabilis* KS-02, and EPF, *T. zelobreve* PBMP16, were obtained from Mycorrhiza and Mycotechnology Laboratory, Department of Microbiology, Khon Kaen University, Khon Kaen, Thailand.

### 2.2. Preparation of AMF Inoculum

AMF inoculum was prepared through the multiplication of AMF spores in pots using maize as a host plant. Maize seeds were surface-sterilized by soaking in a 6% sodium hypochlorite solution for 5 min, rinsed with sterile distilled water, and then sown in plastic pots filled with sterile soil (soil was sterilized in an autoclave at 121 °C for 2 h). AMF inoculum was added to the pots containing the sterilized soil and seeds. The plants were cultivated in a greenhouse and irrigated daily with tap water. After 90 days of planting, irrigation was stopped, and plant roots were collected and examined for root colonization following the method described by Koske and Gemma [19]. The soil samples were air-dried, and AMF spores were quantified using the sucrose centrifugation method [20]. AMF spores were required to have no less than 25 spores g^−1^ soil for use in the experiments.

### 2.3. Preparation of EPF Inoculum

EPF were cultured on potato dextrose agar (PDA) for 7 days. Agar plugs containing the EPF were then transferred into a glass bottle containing sterile sorghum seeds and incubated at room temperature until fungal hyphae fully colonized the sorghum seeds (incubated for approximately 1–2 weeks) [21].

### 2.4. Preparation of Rice Seeds

The cultivar of black upland rice (*Oryza sativa* subsp. *indica*) used in this study, named Maled Phai, was provided by the Rice research project group at the Faculty of Agriculture, Khon Kaen University, Thailand. The rice seeds were surface sterilized by soaking in a 6% sodium hypochlorite solution for 5 min, rinsed with sterile distilled water, then sown in trays containing peat moss, and allowed to grow for 15 days. Then, seedlings were selected for transplantation into pots in the next experiment.

### 2.5. Soil Preparation and Experimental Design

The soil used in the experiment was collected from farmers’ plots and excavated from the surface to a depth of approximately 50 cm. Its chemical and physical properties were analyzed: soil textural class, sandy loam; pH, 5.9; electrical conductivity, 0.14 dS m^−1^; organic matter content, 0.640%; nitrogen, 0.060 mg kg^−1^; phosphorus, 145.29 mg kg^−1^; potassium, 88.01 mg kg^−1^; available phosphorus, 22.70 mg kg^−1^; exchangeable potassium, 50.20 mg kg^−1^; calcium, 790.26 mg kg^−1^; sodium, 29.17 mg kg^−1^; and magnesium, 67.97 mg kg^−1^; field capacity (FC), 12.96%; permanent wilting point (PWP), 2.51%; available water capacity (AWC), 10.45%; and bulk density, 1.46 g cm^−3^. The experimental setup followed a factorial in a randomized complete block design (RCBD), comprising 12 treatments with 9 replications. Each plastic pot (12-inch diameter × 8.5-inch height) was filled with 7.5 kg of non-sterilized soil, and one rice seedling was transplanted per pot. The first factor consisted of three water management levels—100% field capacity (FC), 66% FC, and 33% FC—and the second factor was the addition of AMF and EPF. AMF inoculum was applied at a rate of 200 spores per pot, while EPF inoculum consisted of two EPF-colonized sorghum seeds per pot. Soil moisture was maintained at the field capacity (12.96%) for all treatments during the first 30 days after transplantation. Thereafter, irrigation was adjusted to three designated water regimes. Soil moisture content was monitored daily by collecting soil samples, and irrigation was applied daily to maintain soil water content at 100%, 66%, or 33% of field capacity based on the measured moisture values. The treatment is detailed in Table 1.

### 2.6. Determination of Plant Growth Parameter

After 90 days of transplantation, plant growth parameters were determined, including the plant height and number of tillers. The SPAD values of the plant leaves were measured using a SPAD-502 Plus chlorophyll meter (Spectrum Technologies Inc., USA). The leaf area was measured using an LI-3100C area meter (LI-COR Inc., Lincoln, NE, USA). The chlorophyll contents were analyzed using the method described by Arnon [22]. The photosynthetic rate, stomatal conductance, and transpiration rate were determined using the second expanded leaves from the top of the plants, measured using a portable photosynthesis system (LI-6400XT, LI-COR Inc., Lincoln, NE, USA). Water use efficiency (WUE) was calculated from the photosynthetic rate divided by the transpiration rate. The relative water content (RWC) of the plant was determined using the method described by Suriya-Arunroj et al. [23].

At the harvest stage (120 days after transplant), the number of panicles and seed weight were measured. Plant biomasses (leaves, stems, and roots) were dried at 80 °C for 3 days and then measured, and the dried plant samples were analyzed for nutrient uptake concentration (N, P, K). 

### 2.7. Root Quality Measurement

The root weight was determined. Then, approximately 10% of the roots were sampled and scanned using an Epson Perfection V800 photo scanner (Epson Seiko Epson Corporation, Nagano Prefecture, Suwa, Japan). The scanned data were analyzed to measure root length, size, quantity, and root area using the WinRhizo program (WinRhizo Pro 2004a software, REGENT Instruments Inc., Quebec, QC, Canada).

### 2.8. Assessment of AMF and EPF Root Colonization

To investigate AMF and EPF root colonization frequency, fresh root samples were stained according to the method described by Koske and Gemma [19]. The AMF colonization frequency in stained root segments was observed using Trouvelot’s method [24], and the EPF colonization frequency was described according to Mehmood et al. [25]. The total amount of AMF spores in the soil was determined using the sucrose centrifugation method described by Daniels and Skipper [20].

### 2.9. Plant Biochemical-Related Drought Stress

Reactive oxygen species (ROS) and drought-associated proteins were measured, including superoxide dismutase (SOD), glutathione reductase (GR), peroxidase (POD), ascorbate peroxidase (APX), catalase (CAT), malondialdehyde (MDA), and hydrogen peroxide (H_2_O_2_). Plant leaf samples were ground using liquid nitrogen, and 0.1 g of the resulting material was analyzed using an assay kit from Solarbio (Beijing, China).

Free proline in leaf tissues was extracted and analyzed following a modified method described by Lowry et al. [26]. In short, 0.5 g of grounded plant leaves was added to 10 mL of 3% sulfosalicylic acid and centrifuged at 4000 rpm for 5 min. Then, 2 mL of supernatant was transferred into tubes, and 2 mL of ninhydrin acid reagent and 2 mL of glacial acetic acid were added. Incubated at 100 °C for 1 h, the reaction was terminated by placing the tubes in an ice bath. The mixture was mixed with 4 mL of toluene, and the solution settled into a layer. The top layer was measured for absorbance at 520 nm with a spectrophotometer (U-5100 Hitachi High-Tech Science Corporation, Tokyo, Japan) using L-proline as a calibration standard.

### 2.10. Determination of Functional Substances

The extraction and analysis of phenolic compounds and anthocyanin substances in rice seeds were modified according to the method of Kapcum et al. [27]. Briefly, 1 g of finely ground rice grain samples was added to 10 mL of methanol, and the mixture was shaken in the dark for 2 h. Then, the mixture was centrifuged at 4000 rpm for 10 min. The liquid was filtered with Whatman No. 1 filter paper, and extraction was performed again using the sediment from the previous extraction with 5 mL of methanol solution. Then, the two extracts were mixed well and stored at −40 °C.

The phenolic compounds in the seeds were extracted and analyzed. In total, 125 µL of the extracted sample was taken and mixed with 250 µL of Folin–Ciocalteu reagent solution and 3 milliliters of sterile distilled water, and then incubated at room temperature for 6 min. Thereafter, 2.5 mL of 7% sodium carbonate solution was added, and the mixtures were incubated at room temperature for 90 min. After that, the mixtures were measured for absorbance at a wavelength of 760 nm with a spectrophotometer (U-5100 Hitachi High-Tech Science Corporation, Tokyo, Japan). The absorbance values were compared to a standard curve of gallic acid.

The anthocyanin content was determined using the pH differential method. Briefly, two aliquots of the seed extract, each with a volume of 50 µL, were prepared. The first portion was mixed with 3 mL of 0.025 M potassium chloride solution at pH 1.0, while the second portion was mixed with 3 mL of 0.4 M sodium acetate solution at pH 4.5; then, the mixture was allowed to stand for 20 min before measuring absorbance at 520 nm and 700 nm. Total anthocyanin content was calculated using Equation (1) and expressed as the cyanidin-3-glucoside equivalent per 100 g sample:

Total anthocyanins (mg 100 g^−1^) = (∆A/ϵL) × MW × DF × (V/G) × 100 (1)

where ∆A is absorbance [pH 1.0 (A520 nm–A700 nm)–pH 4.5 (A520 nm–A700 nm)], ϵ is the molar extinction coefficient of Cy-3-G (26,900 M^−1^ cm^−1^), L is the cell path length of cuvette (1 cm), MW is the molecular weight of anthocyanin (449.2 g mol^−1^), DF is the dilution factor, V is the final volume (mL), and G is the weight of sample (g).

DPPH free radical scavenging activity was determined with a method described by Leong and Shui [28] with some modifications. A DPPH solution was prepared with a concentration of 0.05 mM in a methanol solution. A volume of 4.5 mL of DPPH solution was mixed with 100 μL of rice seed extracts and then left to stand at room temperature for 30 min in the dark. The mixtures were measured for absorbance at a wavelength of 517 nm with a spectrophotometer (U-5100 Hitachi High-Tech Science Corporation, Tokyo, Japan) using ethanol as a blank. The percentage of radical scavenging ability was calculated with Equation (2):

% Radical scavenging = [(A_contol_ − A_sample_)/A_control_] × 100 (2)

where A_control_ is the absorbance value of the DPPH solution, and A_sample_ is that of the DPPH solution mixed with rice extract.

### 2.11. Statistical Analysis

Analysis of variance (ANOVA) was conducted using the Statistix 10 software. Data were analyzed according to the factorial in the randomized complete block design (RCBD), and the least significant difference (LSD) test was applied to test for significant differences among the means of different treatments at a *p*-value < 0.05.

## 3. Results

### 3.1. Effect of AMF and EPF on the Growth Promotion of Black Upland Rice Under Drought Conditions

Plants inoculated with EPF and AMF+EPF had significantly higher plant height increase compared to un-inoculated plants and plants inoculated with AMF, under well-watered conditions. While under water-limited conditions (66% and 33%), there was no difference between inoculated and uninoculated cultivation. Under well-watered conditions, the number of rice tillers treated with EPF was highest. Under the water-limited condition of 66%, both individual and co-inoculation promoted a higher number of tillers, but there was no difference compared to the control. Plants inoculated with EPF and AMF+EPF had a significantly higher number of tillers than uncultured plants and plants grown with AMF alone, especially EPF treatment. Shoot fresh weight and dry weight of plants under water-limited conditions (66% and 33%) of inoculated and uninoculated plants showed no significant difference. Under well-watered (100%) conditions, plants inoculated with EPF and AMF+EPF had significantly higher fresh weight than controls, and plants inoculated with AMF and AMF+EPF also had significantly higher dry weight than controls, as shown in Table 2.

The SPAD values and leaf area of inoculated plants compared with the control has no significant difference under well-water and water-limited conditions (66% and 33%) were not significantly different (Table 2). The photosynthetic rate and total chlorophyll of plants inoculated with AMF, EPF, and AMF+EPF were higher than in uninoculated plants under all watering conditions, especially at 33% FC. The transpiration rate of inoculated plants was higher than that of uninoculated plants when cultivated under well-watered and water-limited conditions at 66% FC, but compared with the control, the transpiration rate of the AMF and EPF treatments was not significantly different under water-limited conditions at 33% FC. The stomatal conductance of inoculated and uninoculated plants did not differ under any conditions. Under all conditions, AMF and EPF promoted higher water use efficiency than uninoculated plants, and under water-limited conditions at 33% FC, uninoculated plants showed significantly reduced water use efficiency compared to plants inoculated with AMF, EPF, and AMF+EPF. In addition, the inoculated plants had higher relative water content (RWC) in leaves than uninoculated plants under all conditions. The RWCs of the inoculated plants and control were in the ranges of 84–89% and 80–86%, respectively (Table 3).

After 120 DAT, the rice parameters at the harvesting stage were measured, including the number of panicles, total seed weight, biomass, and nutrient uptake, as shown in Table 4. The number of rice panicles grown with AMF, EPF, and AMF+EPF treatment did not differ significantly compared with the control. The grain weight per plant of inoculated rice tended to be higher than that of the control, especially with co-inoculation. However, at restricted water levels, there was no significant difference between the control and single-inoculation methods. Under normal water conditions, co-inoculated rice showed significantly higher grain weight. Under well-water and water-limited conditions at 66%, plants inoculated with AMF+EPF tended to have the highest biomass, but at 33% water, no difference was found among the treatments. The N value in control plants was significantly higher than in inoculated plants at normal water levels. Furthermore, the *p* value was highest in plants inoculated with AMF, but still not significantly different from the control. Under well-watered conditions, plants inoculated with EPF showed significantly higher potassium (K) content compared with the control. Under water-limited conditions at 66%, plants treated with both fungi tended to increase N and P relative to the control, while the K content was significantly higher in plants inoculated with AMF. The inoculations of AMF and EPF, whether individual and co-inoculation, were found to provide higher N, P, and K levels than uninoculated plants, under water-limited conditions at 33%.

### 3.2. Root Quality

**Figure 1:**
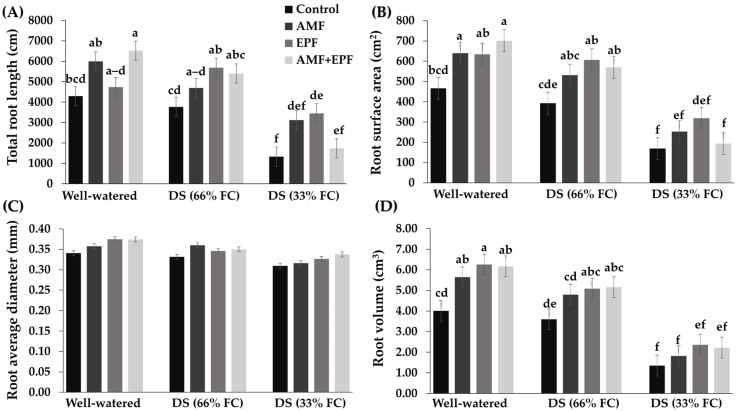
Root length (**A**), root surface area (**B**), root average diameter (**C**), and root volume (**D**) of rice plants under well-watered (WW) and water-limited conditions (WD) of 66% and 33% FC at 90 DAT. Values are means of nine replicates (*n* = 9). Different letters on the bar indicate significant differences in the mean values (*p* ≤ 0.01 using the LSD test).

Figure 1 shows the root growth quality of Maled Phai at the harvesting stage, considering the root length, root surface area, root diameter, root volume, specific root length, and root tissue density. The results indicate that the individual inoculum of either AMF or EPF and their coinoculation significantly enhanced root length, root surface area, and root volume under all drought conditions, which were better than the control treatment. Under well-watered conditions, plants treated with AMF+EPF had the highest root length and surface area, 6511.40 cm and 700.69 cm^2^, respectively, which were significantly higher than the control. Under water-limited conditions of 66% FC, plants inoculated with EPF had the highest root length and root surface area, with similar results under water-limited conditions of 33% FC. However, the root average diameter of each treatment is not different.

### 3.3. Root Colonization

**Figure 2:**
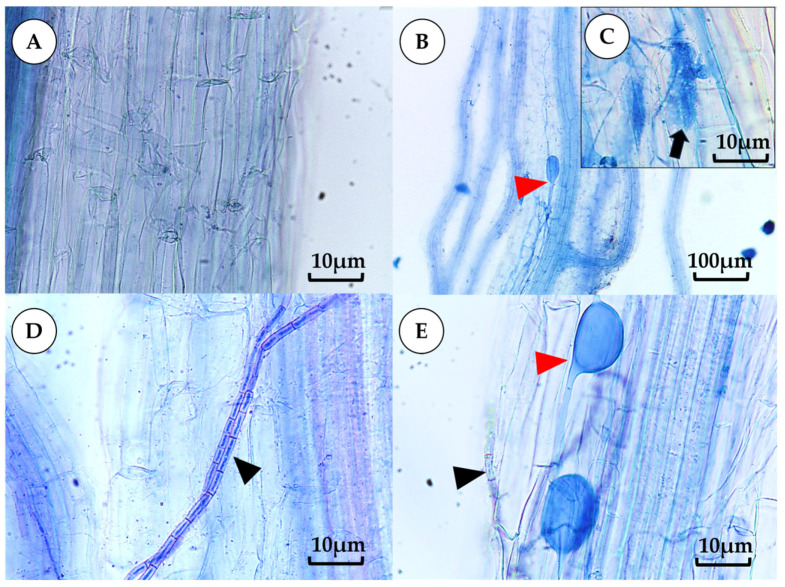
Root colonization of AMF and EPF. Control (**A**); colonization of AMF (**B**,**C**). ((**B**), vesicle of AMF; (**C**), arbuscule of AMF); colonization of EPF (**D**) (black arrowhead, EPF hyphae); colonization of AMF + EPF (**E**) (red arrowhead, vesicle of AMF; black arrowhead, EPF hyphae). All images were obtained using a light microscope. Magnification was 100× for image (**B**) and 400× for images (**A**,**C**,**D**,**E**).

Mycorrhizal fungal structures such as hyphae, arbuscules, and vesicles were observed only in pots of mycorrhizal treatments (Figure 2B,C,F). In contrast, only hyphae were present in pots of EPF inoculum (Figure 2D). Plants inoculated with AMF under well-watered, water-limited at 66% FC, and water-limited at 33% FC conditions showed colonization with AMF at 18.50%, 5.00%, and 9.50%, respectively. The treatments were inoculated with AMF under well-watered, water-limited at 66% FC, and water-limited at 33% FC conditions, exhibiting colonization with EPF at 78.50%, 66.50%, and 86.50%, respectively. Plants inoculated with AMF+EPF showed colonization of both fungal hypha types in their roots. Meanwhile, noninoculated plants showed no colonization of fungi in the roots (Table 5). AMF spores were observed in pots of AMF and AMF+EPF. Spore production is highest, 13.33 spores per gram of soil in AMF treatment under watering 33% FC, which shows no significant difference with treatment AMF+EPF. Similarly, under watering 100% FC, AMF spore production of treatment AMF and AMF+EPF is no different. While under watering 66% FC, AMF treatment produces spores higher than AMF+EPF treatment, but no significant difference, as shown in Table 5.

### 3.4. Determination of Functional Substances

The determination of phenolic compounds, anthocyanins, and antioxidant activity in rice seeds is shown in Table 5. Plants inoculated with AMF+EPF had the highest antioxidant activities of 91.42 under well-watered conditions, and a tendency to be highest under water-limited conditions (66%, 33%). However, there was no significant difference compared to using AMF and EPF individually. Phenolic compounds of plants inoculated with AMF, EPF, and AMF+EPF are higher than the control under well-watered conditions. Plants inoculated with EPF had the highest phenolic compounds under water-limited conditions 33%, while plants inoculated with AMF+EPF had the highest phenolic compounds under water-limited conditions 33%, but no difference compared with plants inoculated with AMF. Anthocyanin of plants inoculated with AMF, EPF, and especially, AMF+EPF treatment was higher than the control under well-watered conditions, and under water-limited conditions (66%, 33%).

### 3.5. Plant Biochemical Contents

The reactive oxygen species and antioxidant enzymes were determined and measured, including CAT, H_2_O_2_, APX, POD, GR, MDA, and SOD (Table 6). Plants inoculated with AMF and EPF had higher CAT, APX, and GR than the control under all drought conditions, exhibiting significant differences compared to the control. In uninoculated plants, POD was higher than in plants inoculated with AMF and EPF when grown under well-watering and water-limited conditions of 66% FC. Proline content in plants inoculated with fungi was higher than in uninoculated plants under all conditions, especially plants inoculated with AMF. Plants inoculated with AMF and EPF had significantly lower H_2_O_2_ contents compared to control plants. In addition, inoculated plants had lower MDA than controls, but there was no significant difference.

The correlation effects of AMF and EPF on plant growth, photosynthetic performance, water use efficiency, yield, seed functional compounds, and antioxidant responses varied depending on water availability. Under well-watered conditions (Table S1), AMF significantly enhanced chlorophyll concentration and water use efficiency, and also positively affected root growth and yield. In addition, AMF promoted the accumulation of proline and anthocyanins; however, it did not significantly influence relative water content (RWC), H_2_O_2_ levels, or phenolic compounds. In contrast, EPF exerted a positive effect on photosynthetic rate, chlorophyll content, RWC, yield, and catalase (CAT) activity under well-watered conditions, and also enhanced the accumulation of phytochemical compounds in rice grains. Under drought conditions (Tables S2 and S3), both AMF and EPF significantly improved antioxidant enzyme activities and markedly reduced H_2_O_2_ accumulation, indicating enhanced oxidative stress tolerance. These results suggest that AMF and EPF contribute to drought resilience through complementary physiological and biochemical mechanisms, with their effects becoming more pronounced under water-limited conditions.

## 4. Discussion

In general, AMF and EPF are beneficial for agriculture. Both types of fungi are classified as plant growth-promoting microbes (PGPMs), and they can also help plants tolerate drought conditions. In this study, the AMF species *R. variabilis* KS-02 and endophytic fungus *T. zelobreve* PBMP16 were selected for use due to their ability to promote plant growth and enhance the accumulation of functional compounds in black upland rice [16,29].

This study demonstrated that the colonization of *R. variabilis* KS-02 and *T. zelobreve* PBMP16 played a role in promoting rice growth in terms of plant height, plant weight, and tillering. This result is similar to those in several previous studies that have shown the ability of AMF and EPF to promote plant growth. For example, the research of Intana et al. [30] reported that *Trichoderma breve* Z2-03, which can produce IAA, can promote seed germination and enhance rice plant growth. Similarly, the research of Kandar et al. [31] reported that EPF can promote rice growth in terms of height, and some research found that some EPF strains are phosphate-solubilizing fungi that can solubilize soil phosphate into a form that plants can use, which helps accelerate plant growth [32]. From the research of Campo et al. [33], AMF can promote plant growth, especially *Rhizophagus irregularis*, which can increase rice growth more than the control. Similarly to the research of Khaekhum et al. [34], which reported that AMF and EPF, as well as their coinoculation, were effective in promoting the growth and tuber yield of *Helianthus tuberosus* L. In addition, this study shows that the growth performances of plants inoculated with AMF and EPF are higher than those of the control, such as in terms of plant height, plant weight, and tillering, under water-imitated conditions at 66% and 33% FC. Similarly, Perumal et al. [35] reported endophytic fungi such as *Colletotrichum spaethianum* AVRF1 and *Gliocladiopsis aquaticus* EHS1 to increase the shoot length and shoot dry weight of soybean under drought conditions, compared to the control.

The analysis of plant photosynthetic rates showed that at normal water levels and under water-limited conditions, plants inoculated with EPF and AMF had higher photosynthetic rates than uninoculated plants, which is consistent with the transpiration rate, leaf area, water use efficiency, and total chlorophyll. Similarly to the research of Wang et al. [36], which reported that EPF can stimulate plant growth and increase the photosynthesis rate and chlorophyll content in leaves, Begum et al. [37] reported that AMF can help plants absorb water and minerals, thus potentially promoting a higher photosynthesis rate than the control and resulting in increased chlorophyll content in the leaves and increased leaf growth. In addition, the RWC values in the leaves of inoculated plants were higher than those of control plants under drought conditions. The RWC value indicates the water status of the plant relative to the water-holding capacity of the leaves. A high RWC value indicates that the plant tends to conserve water in the cell well [38].

This study found that rice inoculated with AMF and EPF had higher yield and biomass than the control under water limitations. Similarly, the research of Ahmadvand and Hajinia [39] reported that endophytic fungus (*Piriformospora indica*) can increase grain yields and biomass of millet under water stress, and AMF and EPF can also help absorb water and nutrients from the soil through the fungal hyphae that grow in the soil around the roots into the plant roots under drought conditions [40,41]. This is consistent with the results showing that AMF- and EPF-inoculated plants accumulated more N, P, and K than uninoculated plants when grown under water-limited conditions.

Our results show that the root growth of plants cultivated with AMF and EPF, such as in terms of root length, root surface area, and root volume, was higher than that of the control. This is consistent with the research of Nacoon et al. [42], who reported that AMF (*R. variabilis* KS-02) and EPF (*T. zelobreve* PBMP16) significantly enhanced the root growth of Maled Phai rice, higher than the control. From the above experimental results, AMF and EPF can be confirmed to promote rice growth through their ability to absorb water and minerals for plants [43], as well as the ability of EPF to produce growth-related hormones and other properties related to plant growth [44].

This study investigated the root infestation of AMF and EPF and found no significant difference under well-watered conditions, whether used individually or in co-culture. When water is restricted, an increase in AMF infestation is observed because under drought conditions, plants produce more of a phytohormone called strigolactone to promote root growth and stimulate AMF inoculation in the root [45]. Meanwhile, EPF in the roots of plants inoculated with AMF+EPF treatment is decreased. Khaekhum et al. [34] observed EPF fungal hyphae in roots two weeks after planting, while AMF were present in very small quantities, possibly indicating that EPF, an endophytic fungus, colonized the roots before AMF, a soil fungus; these two types of fungi share a space to exchange nutrients with plants. However, further research is needed on the interaction of AMF and EPF to understand the mechanisms of their synergy and coexistence in plant roots in the future.

Analysis of anthocyanin, phenolic compounds, and antioxidant activity showed that AMF and EPF increased anthocyanin and achieved the highest antioxidant activity in plants. Plants cultivated with EPF under water-limited conditions at 66% FC increased the maximum phenolic compounds. The results are consistent with previous studies of Nacoon et al. [19] and Gateta et al. [16], who studied the ability of AMF (*R. variabilis* KS-02) and EPF (*T. zelobreve* PBMP16) to increase anthocyanin in Maled Phai rice more than the control and to achieve rice growth similar to that with chemical fertilizers. In addition, Velasco et al. [46] found that the EPF (*T. hamatum*) helped plants increase their accumulation of important functional compounds, including glucosinolates and several compounds with antioxidant activity. Similarly, Lombardi et al. [47] reported that *Trichoderma* sp. strains TH1 and GV41 can increase anthocyanin content in strawberries by 31% and 66%, respectively. Interestingly, with plant cultivation under water-limited conditions at 66% and 33% FC, AMF and EPF also increased the accumulation of anthocyanins, phenolic compounds, and antioxidant activities in most plants compared to the control. However, in this study, rice treated with EPF had the highest accumulation of phenolic compounds in the grains was significantly observed at a water level of 66%.

Regarding the effect of reactive oxygen species in plants, plants inoculated with AMF and EPF achieved higher levels of catalase (CAT) and ascorbate peroxidase (APX) enzymes than uninoculated plants at normal watering levels and tended to achieve higher levels under water-limited conditions. This is consistent with the research of Li et al. [48], who reported that the activities of CAT and APX in plants inoculated with AMF were significantly higher than those in the control when grown under drought conditions. CAT and APX scavenge free radicals, with catalase degrading H_2_O_2_ into H_2_O and O_2_, while APX degrades H_2_O_2_ into H_2_O and dehydroascorbic acid (DHA) using ascorbic acid as a reducing agent [49]. In addition, inoculated plants had a lower concentration of H_2_O_2_, which can reduce cell aging and degradation, than uninoculated plants. Therefore, a lower concentration of H_2_O_2_ reduced the stimulation of the lipid peroxidation process, producing Malondialdehyde (MDA), which damages cell membranes, resulting in reduced photosynthesis and water and nutrient uptake by plants. However, plants inoculated with AMF and EFE showed significantly higher GR content than the control at all watering levels. GR is a potent antioxidant enzyme that functions to maintain glutathione (GSH) status through the ascorbate–glutathione pathway by converting glutathione disulfide (GSSG) to GSH and to protect the cell membrane from oxidation under stress conditions by protecting the thiol groups of proteins. GSH is also a substrate for glutathione-S-transferases (GST) or glutathione peroxidase (GPX), which are involved in the elimination of H_2_O_2_ [50,51]. This experiment also found that the GR enzyme was higher in inoculated plants than in control plants. Similarly to the research of Li et al. [52], rice seedlings inoculated with endophytic fungus EF0801 showed a greater increase in GR than with the control, and there was also an increase in glutathione (GSH) when seedlings were grown in simulated drought conditions with the addition of Polyethylene glycol (PEG) in the nutrients. In addition, inoculated plants had a lower H2O2 concentration than uninoculated plants.

Proline plays an important role in regulating plant survival under drought stress, helps cell signaling processes, and possesses ROS scavenging properties [53,54]. In the present study, rice plants inoculated with AMF and EPF had a higher accumulation of proline amino acids than the control under all watering conditions. Similarly, previous research has shown that planting rice with AMF and EPF results in plants with higher proline content than the control under drought stress [13,52]. AMF affects proline enhancement in plants [37], and proline that accumulates in plants may be important for plant survival under water deficit stress by playing the role of an osmolyte [55].

Overall, our results indicate that the effects of AMF (*R. variabilis* KS-02) and EPF (*T. zelobreve* PBMP16) are not universally synergistic across all traits. Similarly to previous field studies on upland black rice, single inoculation with either *R. valiabilis* or *T. zelobreve* AMF or EPF promoted growth and phytochemical accumulation. It was found that, in some parameters, the use of single inoculants, such as EPF, promoted growth in terms of height, number of tillers, and seed production. In the combination of EPF+AMF, there was enhanced plant root growth as well as an increase in phytochemical accumulation in rice seeds. [56] As well as the research of Chávez et al. [57], who demonstrated that AMF and EPF contribute to drought tolerance through stabilization of photosynthetic efficiency, maintenance of leaf water status, and enhancement of antioxidant defenses. These findings suggest that the benefits of co-inoculation arise from complementary, rather than additive, physiological and biochemical functions, and that the expression of synergy depends on the trait measured and the intensity of water stress. Therefore, AMF and EPF should be considered flexible bioinoculants whose effectiveness is context-dependent, rather than assuming consistent superiority of co-inoculation under all conditions.

## 5. Conclusions

This study demonstrated that the inoculation of AMF (*R. variabilis* KS-02) and EPF (*T. zelobreve* PBMP16) effectively enhanced the growth parameters, yield, and accumulation of functional compounds in upland black rice under drought stress. Post-harvest, rice inoculated with AMF and EPF exhibited improved physiological traits, including photo-synthetic efficiency, relative water content, and nutrient uptake, which contributed to increased biomass and yield compared with the non-inoculated control, particularly under water-limited conditions. Moreover, the functional compounds in rice grains from inoculated treatments exhibited higher levels of phenolic compounds, anthocyanins, and antioxidant activity. These findings highlight the potential of both single inoculating plants with AMF and EPF and co-inoculation can provide beneficial effects, although the magnitude of the response depended on the specific trait and water regime. Overall, these findings highlight the potential of AMF and EPF, applied individually or in combination, as sustainable biofertilizers to enhance productivity and functional quality of upland black rice in drought-prone environments. However, this study only presents a preliminary experiment. Field trials should be conducted to confirm the results before applying to farmers’ upland rice cultivation. 

## Figures and Tables

**Table 1:** Experimental design setup.

Well-Watered (WW 100% FC)	Drought Condition (WD 66% FC)	Drought Condition (WD 33% FC)
T1: Control (non-AMF, EPF)	T5: Control (non-AMF, EPF)	T9: Control (non-AMF, EPF)
T2: AMF	T6: AMF	T10: AMF
T3: EPF	T7: EPF	T11: EPF
T4: AMF+EPF	T8: AMF+EPF	T12: AMF+EPF

**Table 2:** Plant height, number of tillers, shoot fresh weight, shoot dry weight, leaf area, and SPAD values in plants under well-watered (WW) and water-limited conditions (WD) of 66% and 33% FC at 90 DAT.

Water Condition	Treatments	Height (cm)	No. of Tillers	Fresh Weight (g)	Dry Weight (g)	Leaf Area (cm^2^)	SPAD Values
WW	Control	105.67 b	10.50 bc	145.42 bc	35.54 bc	2398.20 abc	37.60
	AMF	108.33 b	13.00 ab	168.36 ab	44.52 a	3022.70 a	39.70
	EPF	120.00 a	13.33 a	173.02 a	42.29 ab	2698.20 ab	38.80
	AMF+EPF	119.33 a	10.50 bc	175.14 a	45.11 a	2720.50 ab	37.70
WD (66% FC)	Control	99.67 b	9.50 cd	126.45 c	31.66 c	1990.30 cd	35.80
	AMF	105.50 b	12.00 abc	139.83 c	34.26 c	2366.90 bc	38.20
	EPF	100.50 b	11.67 abc	132.69 c	32.96 c	1959.40 cd	37.60
	AMF+EPF	107.00 b	10.00 cd	143.42 bc	36.71 bc	2379.70 bc	36.30
WD (33% FC)	Control	84.50 c	6.67 e	68.45 d	18.70 d	1142.20 e	34.90
	AMF	88.33 c	7.67 de	71.00 d	18.96 d	1058.20 e	36.50
	EPF	85.67 c	10.00 cd	83.35 d	20.25 d	1574.10 de	37.70
	AMF+EPF	83.50 c	9.67 cd	89.84 d	24.07 d	1569.70 de	35.70
% CV		6.59	15.62	12.47	13.53	18.23	4.54
F-test		**	*	**	**	**	ns

**, significant difference at *p*-value ≤ 0.01; *, significant difference at *p*-value ≤ 0.05; ns, no significant difference. Values are means of nine replicates (*n* = 9). Different letters indicate significant differences among values in the same column based on the LSD test.

**Table 3:** Photosynthetic rate (Pn), stomatal conductance (Sc), transpiration rate (Tr), water use efficiency (WUE), relative water content (RWC), and total chlorophyll (Chl) in plants under well-watered (WW) and water-limited conditions (WD) of 66% and 33% FC at 90 DAT.

Water Condition	Treatments	Pn (µmol CO_2_ m^−2^ s^−1^)	Sc (H_2_O m^−2^ s^−1^)	Tr (mmol H_2_O m^−2^ s^−1^)	WUE (µmol CO_2_ mmol H_2_O m^−2^ s^−1^)	% RWC	Chl mg·L^−1^
WW	Control	4.3 de	0.41	1.63 de	2.64 a–d	86.49 abc	62.50 bc
	AMF	4.5 de	0.08	1.29 e	3.66 a	88.39 abc	63.09 abc
	EPF	9.3 ab	0.17	3.37 abc	2.78 abc	89.85 a	64.00 ab
	AMF+EPF	10.4 a	0.20	3.49 ab	3.07 ab	88.39 ab	64.54 a
WD (66% FC)	Control	4.1 de	0.19	2.51 bcd	1.73 d	84.47 bcd	59.90 ef
	AMF	7.7 bc	0.70	3.70 a	1.98 cd	86.53 abc	61.73 cde
	EPF	6.8 bcd	0.14	3.22 abc	2.29 bcd	88.02 abc	61.94 cd
	AMF+EPF	8.0 abc	0.21	3.96 a	2.06 bcd	87.61 abc	63.38 abc
WD (33% FC)	Control	0.4 f	0.09	2.20 cde	0.18 e	80.69 d	58.40 f
	AMF	5.6 cde	0.12	2.37 b–e	2.46 bcd	84.16 cd	60.60 de
	EPF	3.3 e	0.07	1.81 de	1.97 cd	84.72 bcd	62.46 bc
	AMF+EPF	4.2 de	0.05	1.65 de	2.58 bcd	84.04 cd	61.79 cd
% CV		28.08	173.76	26.88	26.56	3.00	1.76
F-test		**	ns	**	**	*	**

**, significant difference at *p*-value ≤ 0.01; *, significant difference at *p*-value ≤ 0.05; ns, no significant difference. Values are means of nine replicates (*n* = 9). Different letters indicate significant differences among values in the same column based on the LSD test.

**Table 4:** Number of panicles, total seed weight, biomass, and nutrient uptake of rice plants under well-watered (WW) and water-limited conditions (WD) of 66% and 33% FC at 120 DAT.

Water Condition	Treatments	No. of Panicles	Total Seed Weight (g·Plant^−1^)	Biomass (g)	Nutrient Uptake mg·g^−1^		
					N	P	K
WW	Control	9.00	13.29 cd	60.19 bc	0.75 ab	0.06 bcd	1.29 i
	AMF	11.00	15.07 c	69.16 ab	0.46 c	0.08 b	1.22 j
	EPF	10.33	19.59 b	68.13 ab	0.43 c	0.05 cd	1.48 d
	AMF+EPF	11.00	24.93 a	72.78 a	0.39 c	0.04 d	1.32 h
WD (66% FC)	Control	8.33	9.69 d–g	52.44 cd	0.50 c	0.05 cd	1.41 f
	AMF	9.67	10.81 def	62.31 abc	0.52 c	0.07 bc	1.57 b
	EPF	10.00	10.10 def	61.10 abc	0.43 c	0.08 b	1.37 g
	AMF+EPF	10.00	11.72 cde	64.36 ab	0.56 bc	0.05 cd	1.23 j
WD (33% FC)	Control	7.33	3.78 h	33.46 e	0.75 ab	0.08 b	1.31 hi
	AMF	8.33	7.55 fgh	35.93 e	0.84 a	0.12 a	2.13 a
	EPF	9.00	5.81 gh	40.28 e	0.94 a	0.13 a	1.45 e
	AMF+EPF	8.67	7.76 e–h	44.01 de	0.82 a	0.11 a	1.54 c
% CV		15.89	20.95	12.63	21.14	21.15	0.91
F-test		ns	**	**	**	**	**

**, significant difference at *p*-value ≤ 0.01; ns, no significant difference. Values are means of nine replicates (*n* = 9). Different letters indicate significant differences among values in the same column based on the LSD test.

**Table 5:** Root colonization of AMF and EPF, AMF spores in soil, antioxidants, total phenolic content (TPC), and total anthocyanin content (TAC) under well-watered (WW) and water-limited conditions (WD) of 66% and 33% FC at 120 DAT.

Water Condition	Treatments	%Root Colonization		AMF Spores. g^−1^ Soil	Antioxidants (%Radical Scavenging)	TPC (mg Gallic Equation 100 g^−1^)	TAC (mg Cy-3-G Equation 100 g^−1^)
		AMF	EPF				
WW	Control	0.00 d	0.00 c	0.00 c	88.38 ab	328.27 de	71.81 bcd
	AMF	18.50 a	0.00 c	8.60 ab	86.86 b	345.00 bcd	72.64 bcd
	EPF	0.00 d	78.50 a	0.00 c	86.00 bc	362.69 ab	76.48 bc
	AMF+EPF	17.50 a	71.50 a	8.07 ab	91.42 a	347.69 bc	85.83 a
WD (66% FC)	Control	0.00 d	0.00 c	00.00 c	86.24 bc	336.35 cde	64.62 d
	AMF	5.00 c	0.00 c	11.87 ab	82.57 c	374.87 a	73.81 bcd
	EPF	0.00 d	66.50 a	0.00 c	87.37 b	332.69 cde	78.65 ab
	AMF+EPF	6.00 c	40.00 b	5.07 bc	87.68 b	336.03 cde	79.15 ab
WD (33% FC)	Control	0.00 e	0.00 c	00.00 c	85.19 bc	301.35 f	49.60 e
	AMF	9.50 b	0.00 c	13.33 a	86.43 b	331.15 cde	69.13 cd
	EPF	0.00 d	86.50 a	00.00 c	87.02 b	323.65 e	65.63 d
	AMF+EPF	10.50 b	63.50 ab	13.00 a	87.84 ab	344.42 cd	71.64 bcd
% CV		26.09	31.99	82.21	2.53	3.15	7.62
F-test		**	**	**	*	**	**

**, significant difference at *p*-value ≤ 0.01; *, significant difference at *p*-value ≤ 0.05. Values are means of nine replicates (*n* = 9). Different letters indicate significant differences among values in the same column based on the LSD test.

**Table 6:** Catalase (CAT), hydrogen peroxide (H_2_O_2_), ascorbate peroxidase (APX), peroxidase (POD), glutathione reductase (GR), malondialdehyde (MDA), superoxide dismutase (SOD), and proline under well-watered (WW) and water-limited conditions (WD) of 66% and 33% FC at 90 DAT.

Water Condition	Treatments	CAT (U g Tissue^−1^)	H_2_O_2_ (µmol g Tissue^−1^)	APX (U g Tissue^−1^)	POD (U g Tissue^−1^)	GR (U g Tissue^−1^)	MDA (nmol g Tissue^−1^)	SOD (U g Tissue^−1^)	Proline (µM g Tissue^−1^)
WW	Control	108.52 de	18.09 b	0.58 c	1319.30 a	0.009 e	67.85	114.11	5.94 c
	AMF	391.12 a–d	14.25 d	3.73 ab	1106.00 ab	0.091 a–d	60.54	119.60	8.03 abc
	EPF	397.90 abc	11.79 f	1.10 c	1104.30 ab	0.056 b–e	60.86	113.41	6.53 c
	AMF+EPF	590.07 ab	16.16 c	3.73 ab	792.33 bcd	0.112 ab	60.86	113.13	7.01 bc
WD (66% FC)	Control	94.95 e	19.42 a	0.79 c	894.00 bc	0.014 e	67.99	114.81	6.40 c
	AMF	644.33 a	16.03 c	1.14 c	676.00 cde	0.045 b–e	64.57	113.64	9.85 a
	EPF	321.04 b–e	8.26 g	2.57 bc	719.00 cde	0.024 de	63.55	110.23	7.46 abc
	AMF+EPF	339.12 b–e	19.40 a	2.68 bc	527.33 de	0.075 a–e	61.83	110.88	6.86 bc
WD (33% FC)	Control	280.34 cde	19.91 a	0.76 c	749.67 bcd	0.030 cde	69.97	112.69	6.54 c
	AMF	300.69 cde	12.05 f	1.02 c	789.00 bcd	0.142 a	65.91	112.51	9.15 ab
	EPF	560.68 abc	12.86 e	2.12 bc	552.67 cde	0.103 abc	66.77	112.80	6.98 bc
	AMF+EPF	465.73 abc	14.17 d	5.89 a	376.33 e	0.045 b–e	66.88	112.85	7.95 abc
% CV		25.55	24.01	26	27.12	21.95	10.94	14.75	19.46
F-test		**	*	**	**	*	ns	ns	*

**, significant difference at *p*-value ≤ 0.01; *, significant difference at *p*-value ≤ 0.05; ns, no significant difference. Values are means of nine replicates (*n* = 9). Different letters indicate significant differences among values in the same column based on the LSD test.

## Data Availability

The datasets obtained and analyzed in the current study are available from the corresponding author upon reasonable request.

## Supplementary Materials

The following supporting information can be downloaded at: https://www.mdpi.com/article/10.3390/jof12010002/s1. Table S1: Correlation between AMF and EPF root colonization, plant growth performance, root length, reactive oxygen species, and antioxidant enzymes related to drought stress, and phytochemicals of rice under well-watered conditions at 100% FC. Table S2: Correlation between AMF and EPF root colonization, plant growth performance, root length, reactive oxygen species, and antioxidant enzymes related to drought stress, and phytochemicals of rice under water-limited conditions at 66% FC. Table S3: Correlation between AMF and EPF root colonization, plant growth performance, root length, reactive oxygen species, and antioxidant enzymes related to drought stress, and phytochemicals of rice under water-limited conditions at 33% FC.
