# Supplementary material for: Promoting Growth Performances and Phytochemicals of Black Upland Rice Through the Co-Inoculation of Arbuscular Mycorrhizal Fungi and Endophytic Fungi Under Drought Conditions
Source: J Fungi (Basel). 2025 Dec 19;12(1):2. doi: 10.3390/jof12010002 (PMC12843334; doi:10.3390/jof12010002)
Supplement: Supplementary file 1 [file jof-12-00002-s001.zip › jof-4001083-supplementary.pdf]

Supplementary Table S1. Correlation between AMF and EPF root colonization, plant growth performance, root length, reactive oxygen species, and antioxidant enzymes related to drought stress, and phytochemicals of rice under well-watered conditions at 100% FC.

	AMF	EPF	Pn	Chl	WUE	RWC	Root length	Biomass	Yield	CAT	H_2O_2	APX	Proline	Antioxidant	TPC
EPF	-0.07ns														
Pn	0.09ns	0.93**													
Chl	0.25*	0.62**	0.66**												
WUE	0.57**	-0.05ns	-0.04ns	-0.03ns											
RWC	0.05ns	0.52**	0.24ns	0.12ns	0.34*										
Root length	0.88**	0.24ns	0.34*	0.38*	0.52**	0.29ns									
Biomass	0.35*	0.13ns	0.33*	0.78**	-0.03ns	-0.35*	0.23ns								
Yield	0.33*	0.78**	0.87**	0.62**	0.14ns	0.31ns	0.64**	0.30ns							
CAT	0.68**	0.67**	0.76**	0.66**	0.38*	0.33*	0.79**	0.44**	0.80**						
H_2O_2	0.04ns	-0.40*	-0.30ns	-0.36*	0.05ns	-0.24ns	-0.04ns	-0.26ns	-0.16ns	-0.35*					
APX	0.97**	0.07ns	0.20ns	0.30ns	0.71**	0.17ns	0.89**	0.32ns	0.41**	0.75**	0.03ns				
Proline	0.54**	-0.02ns	0.04ns	-0.08ns	0.78**	0.26ns	0.49**	-0.05ns	0.14ns	0.37*	0.11ns	0.68**			
Antioxidant	0.64**	0.54**	0.68**	0.55**	0.16ns	0.15ns	0.78**	0.32ns	0.80**	0.83**	0.10ns	0.65**	0.14ns		
TPC	0.03ns	0.55**	0.51**	0.65**	-0.12ns	0.15ns	0.11ns	0.53**	0.41**	0.51**	-0.86**	0.05ns	-0.21ns	0.16ns	
TAC	0.37*	0.65**	0.75**	0.51**	0.07ns	0.24ns	0.58**	0.24ns	0.82**	0.72**	-0.06**	0.40**	0.08ns	0.85**	0.30ns

**, Significant difference at $P < 0.01$; *, Significant difference at $P < 0.05$; ns, non-significant difference

Supplementary Table S2. Correlation between AMF and EPF root colonization, plant growth performance, root length, reactive oxygen species, and antioxidant enzymes related to drought stress, and phytochemicals of rice under water-limited conditions at 66% FC.

	AMF	EPF	Pn	Chl	WUE	RWC	Root length	Biomass	Yield	CAT	H2O2	APX	Proline	Antioxidant	TPC
EPF	-0.08ns														
Pn	0.59**	0.25ns													
Chl	0.54**	0.45**	0.50**												
WUE	0.20ns	0.36*	0.61**	0.43**											
RWC	0.29ns	0.53**	0.65**	0.63**	0.68**										
Root length	0.07ns	0.56**	0.27ns	0.72**	0.29ns	0.53**									
Biomass	0.47**	0.33*	0.54**	0.45**	-0.10ns	0.21ns	0.32ns								
Yield	0.31ns	0.08ns	0.32ns	0.56**	0.58**	0.41**	0.26ns	-0.26ns							
CAT	0.60**	-0.10ns	0.49**	0.40*	0.11ns	0.30ns	0.26ns	0.54**	0.15ns						
H2O2	0.42**	-0.63**	-0.17ns	-0.10ns	-0.35*	-0.41**	-0.48**	-0.11ns	0.04ns	-0.18ns					
APX	0.27ns	0.81**	0.22ns	0.55**	0.15ns	0.59**	0.56**	0.43**	-0.04ns	0.05ns	-0.26ns				
Proline	0.42**	-0.23ns	0.22ns	0.05ns	-0.07ns	0.10ns	0.04ns	0.39*	-0.08ns	0.86**	-0.08ns	-0.02ns			
Antioxidant	0.53**	0.41**	0.59**	0.66**	0.36*	0.58*	0.64**	0.55**	0.09ns	0.66**	-0.43**	0.52**	0.34*		
TPC	0.42**	-0.53**	0.24ns	-0.13ns	-0.07ns	-0.06ns	-0.20ns	0.27ns	-0.11ns	0.78**	0.10ns	-0.36*	0.81**	0.27ns	
TAC	0.29ns	0.59**	0.24ns	0.65**	0.19ns	0.51**	0.82**	0.38*	0.33*	0.37*	-0.32ns	0.69**	0.24ns	0.56**	-0.02ns

**, Significant difference at P < 0.01; *, Significant difference at P < 0.05; ns, non-significant difference

Supplementary Table S3. Correlation between AMF and EPF root colonization, plant growth performance, root length, reactive oxygen species, and antioxidant enzymes related to drought stress, and phytochemicals of rice under water-limited conditions at 33% FC.

	AMF	EPF	Pn	Chl	WUE	RWC	Root length	Biomass	Yield	CAT	H2O2	APX	Proline	Antioxidant	TPC
EPF	-0.10ns														
Pn	0.70**	0.14ns													
Chl	0.24ns	0.63**	0.48**												
WUE	0.70**	0.39*	0.72**	0.64**											
RWC	0.17ns	0.34*	0.35*	0.23ns	0.60**										
Root length	-0.03ns	0.26ns	0.43**	0.55**	0.56**	0.42**									
Biomass	0.31ns	0.59**	0.29ns	0.03ns	0.35*	0.31ns	-0.14ns								
Yield	0.76**	0.21ns	0.65**	0.56**	0.81**	0.44**	0.31**	0.23ns							
CAT	-0.12ns	0.99**	0.17ns	0.67**	0.43**	0.38*	0.38*	0.53**	0.21ns						
H2O2	-0.49**	-0.40**	-0.75**	-0.66**	-0.85**	-0.43**	-0.75**	-0.26ns	-0.73**	-0.46**					
APX	0.54**	0.60**	0.27ns	0.40**	0.53**	0.29ns	-0.26ns	0.61**	0.47**	0.54**	-0.21ns				
Proline	0.74**	-0.21ns	0.78**	0.05ns	0.63**	0.44**	0.22ns	0.22ns	0.48**	-0.18ns	-0.52**	0.16ns			
Antioxidant	0.66**	0.11ns	0.59**	0.43**	0.78**	0.22ns	0.53**	0.17ns	0.64**	0.15ns	-0.68**	0.31ns	0.42**		
TPC	0.72**	0.40**	0.69**	0.33*	0.74**	0.27ns	0.15ns	0.66**	0.57**	0.40*	-0.64**	0.65**	0.57**	0.63**	
TAC	0.64**	0.42**	0.88**	0.49**	0.76**	0.44**	0.36*	0.49**	0.69**	0.44**	-0.76**	0.53**	0.61**	0.63**	0.84**

**, Significant difference at $P < 0.01$; *, Significant difference at $P < 0.05$; ns, non-significant difference
